# From glia limitans to glial scars: in vitro co-culture studies of the astrocyte and meningeal interaction

**DOI:** 10.1186/s12987-025-00715-z

**Published:** 2025-10-21

**Authors:** Erin C. Reardon, Aisling J. Greaney, John J. E. Mulvihill

**Affiliations:** 1 Bernal Institute, University of Limerick, Castletroy, Limerick, Ireland; 2 School of Engineering, University of Limerick, Castletroy, Limerick, Ireland

**Keywords:** Brain-meninges interface, Co-culture model, in vitro systems, Leptomeninges, Pial-glial basement membrane

## Abstract

The brain-meninges interface, comprising of astrocytes and meningeal cells seperated by a shared basement membrane, plays critical roles in the central nervous system. Recent work has shown the importance of signalling between the brain and the meninges in neurodevelopment, health, disease, and in stem cell migration. Despite this recent research, the brain-meninges interface is significantly understudied. This systematic review evaluates 27 studies which examine astrocyte-meningeal cell co-culture models. The papers were identified from a search of PubMed, Scopus, and Web of Science and screened for eligibility according to the PRISMA guidelines. These papers utilized the astrocyte-meningeal cell co-culture to mimic different biological interfaces within the central nervous system such as the spinal cord, optic nerve, and the brain-meninges interface to examine various outcomes such as neurite outgrowth, morphology, glial scar formation, and protein expression. Our findings highlight significant gaps in our understanding of the brain-meninges interface, along with inconsistencies in methodologies when establishing the astrocyte-meningeal cell co-culture model. Finally, this review recommends a standardisation of methodologies for astrocyte-meningeal cell co-culture including model validation and detailed protocols. This will allow for improved understanding of these important interfaces in the brain.

**Supplementary Information:**

The online version contains supplementary material available at 10.1186/s12987-025-00715-z.

## Introduction

The boundary between the brain and the meninges, often referred to as the pial-glial membrane [1, 2], or the brain-meninges interface, plays a crucial role in maintaining central nervous system (CNS) homeostasis as a selectively permeable barrier [3, 4]. The cortical meninges tightly adhere to the brain, and comprises of three main layers – the dura, arachnoid, and pia mater [5]. Initially, the meninges were thought to only provide structural and protective support to the brain [6]. However, recent literature shows that the meninges has a far more complex role within the CNS [7, 8]. They function as a stem cell niche [9], provide immune support to the brain harbouring a diverse cell population [10, 11], and contribute to early neurodevelopment [12, 13]. Despite being an important physiological barrier, the brain-meninges interface still remains understudied [3] with questions remaining on the strucuture and composition of this barrier, and the role the resident cells provide [8].

At a cellular level, the interface between brain and meninges consists of a monolayer of pial cells which line the brain parenchyma [8, 14]. Adjacent to this lies the glia limitans, a specialised layer of astrocytes, which form a critical brain border protecting the brain from external insults [15, 16]. A shared basement membrane lies between these two cell types which is rich in extracellular matrix (ECM) proteins, overall forming the brain-meninges interface [3, 17]. The bi-directional cellular and molecular communication between the meninges and the brain is crucial in health and disease, such as traumatic brain injury (TBI) [18], Alzheimer’s disease [19, 20], and meningitis [7, 21, 22]. Meningeal cells secrete chemokines such as CXCL12 and instructive factors such as retinoic acid in early brain development which is essential for cortical neuron migration and laminar organisation [13, 23, 24].

The diverse immune cell population within the meninges, such as border associated macrophages, can modulate and exacerbate the neuroinflammatory response in injury and disease through immune cell activation [18, 25]. Furthermore, disruption to the meningeal lymphatics can promote the deposition of amyloid beta in Alzheimer’s disease [26]. However, our understanding of how this cellular and tissue barrier mediates bi-directional communication remains limited restricting our ability to study its role in neurodevelopment, health, and disease [27] using the current in vitro methods or in vivo animal models.

Unfortunately, there is limited access to human meningeal tissue and small animal models do not fully replicate that of the human meninges [8]. This is due to various factors such as size, thickness, and structure. Firstly, the human meninges are much larger and thicker than in rodents [28]. There are various structural differences including the presence of trabeculae in the subarachnoid space of human and rats, but not in mice [4, 29]. Furthermore, arachnoid granulations are primarily described in adult humans and larger animals, but not in rodents or human neonates [30, 31]. However, the meningeal cell transcriptome has been shown to have strong molecular identity and conservation of the gene markers across human and mouse [10]. Therefore, it is important to understand both the differences and similarities of the meninges across species. As it is difficult to study the human meninges aside from using post-mortem tissue, a representative in vitro model is required to accurately mimic and use as a platform to study how the brain and the meninges interact. There is a drive to reduce the use of animal models in research and improving the relevancy of in vitro models is a key priority [32]. Co-culturing two or more cell types together has been shown to greatly improve the representation of various in vitro models to a more in vivo state [33, 34], along with contributing to the maturation of cells such as astrocytes [33]. Therefore, a co-culture model using astrocytes and meningeal cells is needed to investigate open questions regarding the brain-meninges interface such as an alternative route for drug delivery, understanding brain-meninges bi-directional signalling, and the role of its resident cells in health and disease [35–37].

The astrocyte-meningeal interaction has also been investigated to model the glial scar or in the reforming of the glia limitans following injury [38–40]. In particular, it has been used to model penetrating injuries, where meningeal cells invade the brain parenchyma, and astrocytes seal off the lesion, reforming the glia limitans [41, 42]. This has a dual role within the CNS. The glial scar can act as a protective mechanism to prevent the further spread of damage [43]. However, it can also have detrimental effects preventing neurite outgrowth and tissue repair [44–47]. The mechanism behind this is not fully understood and requires further study [15].

Overall, astrocyte-meningeal co-culture models have been used to model various interfaces in the CNS including the glial scar [39], optic nerve [48], spinal cord [38], and brain-meninges interface [49]. However, there is limited literature which focuses on the methods required to create an in vitro model that replicates the brain-meninges interface through co-culturing of astrocytes and meningeal cells. By clarifying the experimental methods, key parameters, and functional outputs used in these models, this systematic review will highlight existing gaps in knowledge and provide direction to develop an in vitro model of the brain-meninges interface. This systematic review will compare and critically evaluate the methodologies and functional assessments of astrocyte-meningeal co-culture models.

## Methods

There is currently no consensus on the terminology for the resident cells of the meninges that are adjacent to the brain. The term that has commonly been used is “meningeal fibroblast cells” [8]. Consequently, the literature uses various terms such as meningeal cells, fibroblasts, pial cells, arachnoid cells, meningothelial cells and so on. For purpose of this review, these cells will be referred to as meningeal cells, as this term broadly encompasses the majority of the studies reviewed and includes cells which may be derived from the dura, arachnoid, or pia mater. However, this review is not an attempt to standardise or create a consensus on the terminology of this cell type. Furthermore, this review does not aim to examine in vivo interactions but focuses on the current state of in vitro models of the astrocyte-meningeal interface.

The Preferred Reporting Items for Systematic Reviews and Meta-Analyses (PRISMA) process was followed in this systematic review to identify and select the papers reviewed:

- Scopus search string: (TITLE-ABS-KEY (“meningeal cells” OR “meningothelial cells” OR “pia mater cells” OR “arachnoid cells” OR “dura mater cells” OR “leptomeninges” OR “leptomeningeal cells” OR “meningeal cells” OR “fibroblast”) AND (“astrocytes” OR “astroglial cells” OR “glial cells”) AND (“co-culture” OR “coculture” OR “brain meninges interface” OR “glial scar” OR “communication” OR “glia limitans”).
- Web of Science search string: All Fields (“meningeal cells” OR “meningothelial cells” OR “pia mater cells” OR “arachnoid cells” OR “dura mater cells” OR “leptomeninges” OR “leptomeningeal cells” OR “meningeal cells” OR “fibroblast”) AND (“astrocytes” OR “astroglial cells” OR “glial cells”) AND (“co-culture” OR “coculture” OR “brain meninges interface” OR “glial scar” OR “communication” OR “glia limitans”).
- PubMed search string: (“meningeal cells” OR “meningothelial cells” OR “pia mater cells” OR “arachnoid cells” OR “dura mater cells” OR “leptomeninges” OR “leptomeningeal cells” OR “meningeal cells” OR “fibroblast”) AND (“astrocytes” OR “astroglial cells” OR “glial cells”) AND (“co-culture” OR “coculture” OR “brain meninges interface” OR “glial scar” OR “communication” OR “glia limitans”).
- Search was completed using the default search fields.
- Search was limited to articles published in English.

The identified studies were then screened based on the inclusion and exclusion criteria for the systematic review.

### Inclusion criteria

- Articles had to co-culture meningeal cells and astrocytes to be included in this systematic review.
- Must be an original research article.

### Exclusion criteria

- Papers were also excluded if they only used conditioned media as their co-culture method.
- Papers using explants were not included in this systematic review due there being a heterogeneous population of cells included.
- The meningeal “fibroblast” cells must be derived from the cortex, spinal cord, or optic nerve. Any papers using skin or other types of fibroblasts were excluded.

**Fig. 1:**
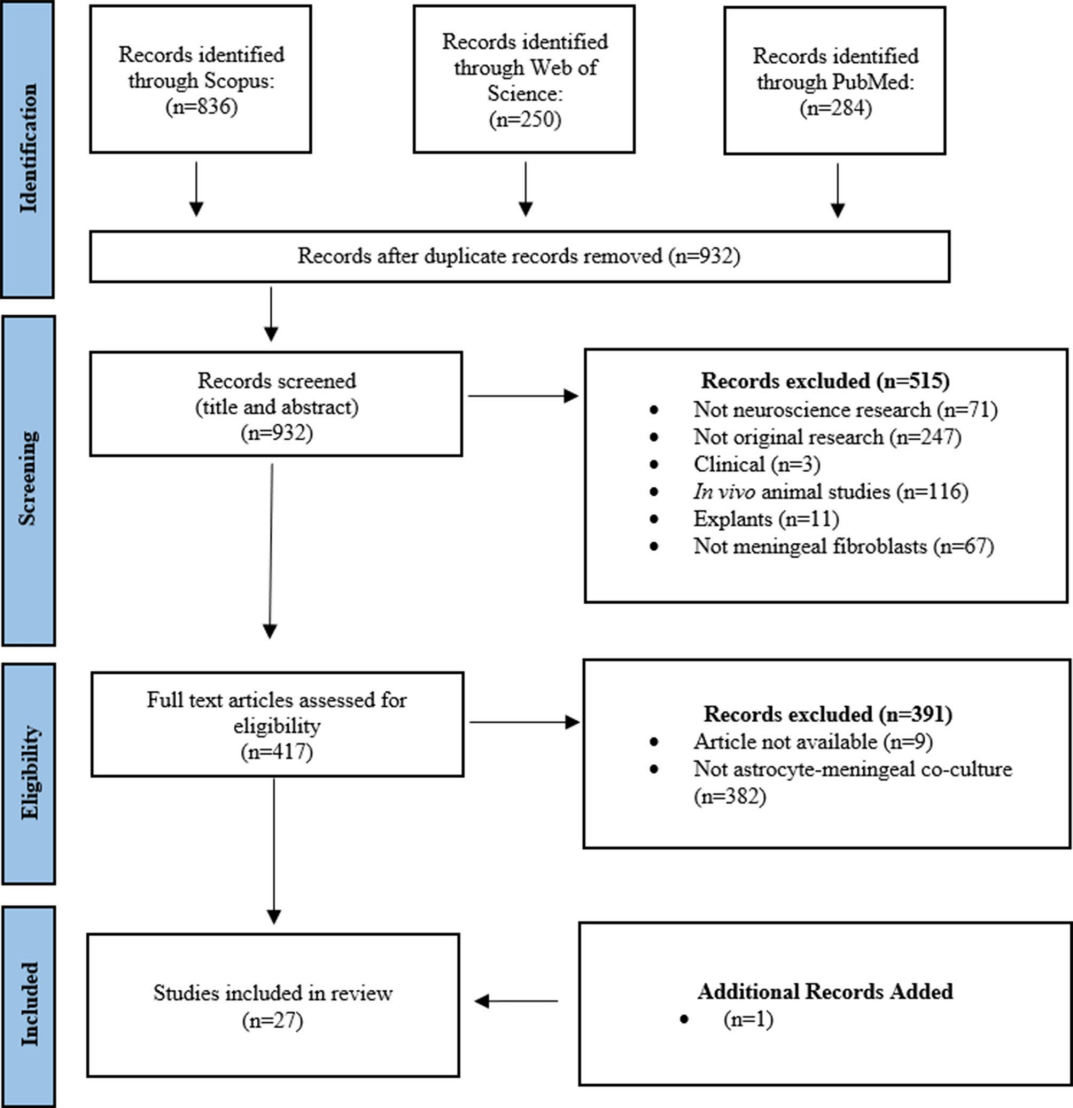
Workflow diagram summarizing the identification of articles utilizing three separate search platforms; Scopus, Web of Science, and PubMed. One additional paper was identified outside of the search string which was included in the systematic review due to meeting the outlined criteria

The PRISMA flowchart in Fig. 1 details the papers which were identified and excluded for this systematic review.

## Results

In total, 27 papers were selected which co-cultured astrocytes and meningeal cells in vitro. In this systematic review, the co-culture methods, parameters, and outputs were gathered and discussed. For the presented systematic review, the papers needed to focus on, or detail, the development of the co-culture model using astrocytes and meningeal cells. In some cases, the co-culture of astrocytes and meningeal cells were used as an experimental condition with little details provided. These studies were still included as they may have some useful information regarding the co-culturing of astrocytes and meningeal cells.

### Objectives for examining astrocyte-meningeal co-cultures

**Fig. 2:**
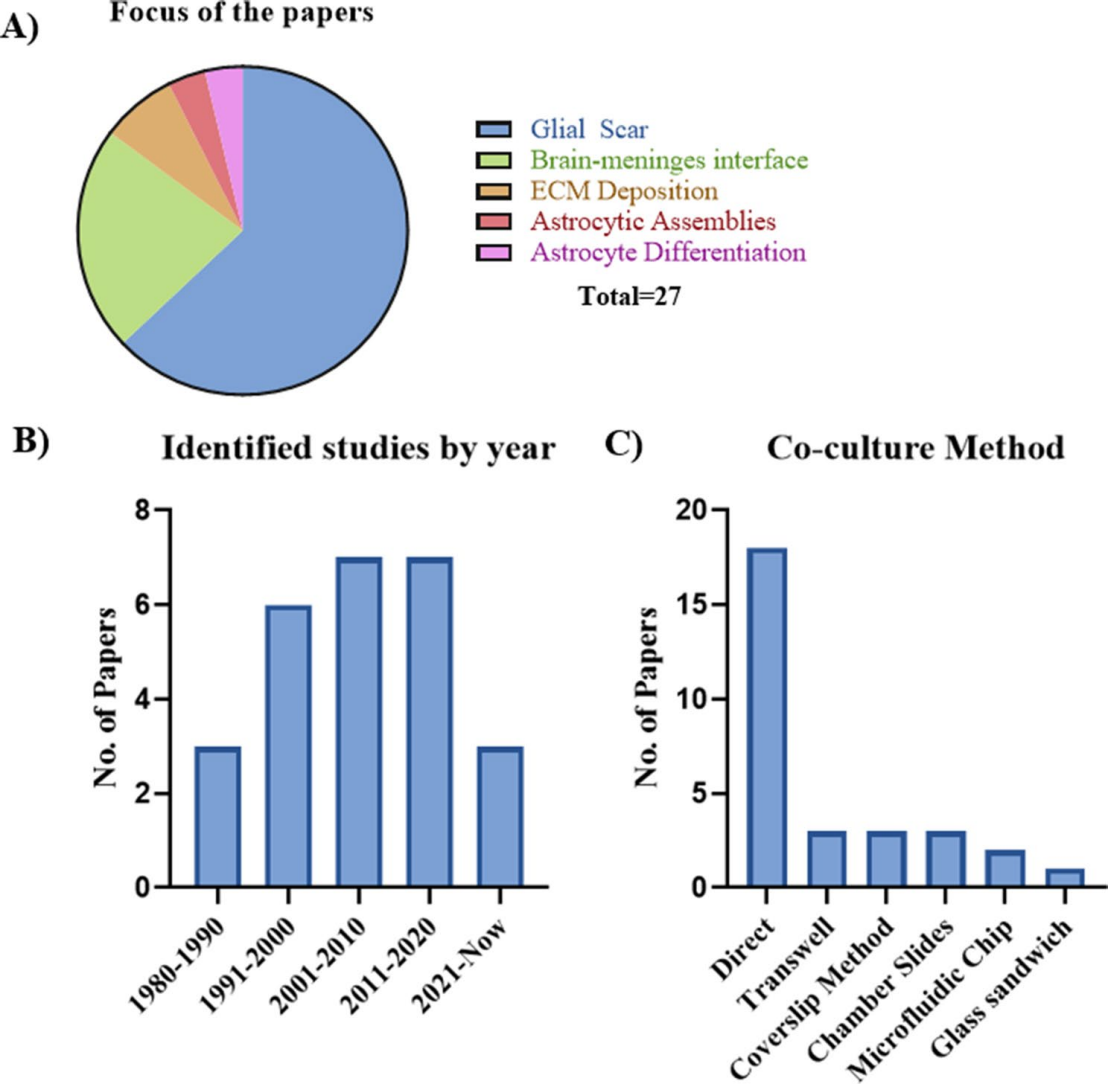
**A**) Breakdown of the main focus of each of the reviewed papers. Glial scar (17/27), brain-meninges interface (6/27), ECM deposition (2/27), astrocytic assemblies (1/27), and astrocyte differentiation (1/27). **B**) Summary of the papers identified by year. **C**). Summary of the co-culture method used in the reviewed papers with some papers using more than one method. Direct (18/27), Transwell/Millicell (3/27), direct using coverslip method (3/27), chamber slides (3/27), microfluidic chip (2/27), and glass sandwich (1/27). Graphs made using GraphPad Prism Version 10.4.1

The included papers had different research questions involving this co-culture model (Fig. 2). Most of the papers focus on the formation of the glial scar following injury, with a particular focus on its inhibitory effects on neurite outgrowth and axonal regeneration (17/27). Whereas six studies (6/27) explored the interface between the brain and meninges at the cellular level, often focusing on the development of the glia limitans. The remaining papers (4/27) had more focused research questions. These studies explored tenascin deposition in the optic nerve, collagen deposition, astrocyte differentiation due to co-culture, and astrocytic assemblies. Therefore, the review will now detail the methods and parameters used to set up the co-culture model rather than the motivation or purpose of the studies.

### Co-culture methods

The details of the co-culture model set up including cell type, seeding density, co-culture model type, coatings, etc. were collected from all 27 papers and are included and referenced in the supplementary file.

#### Cell types used

**Fig. 3:**
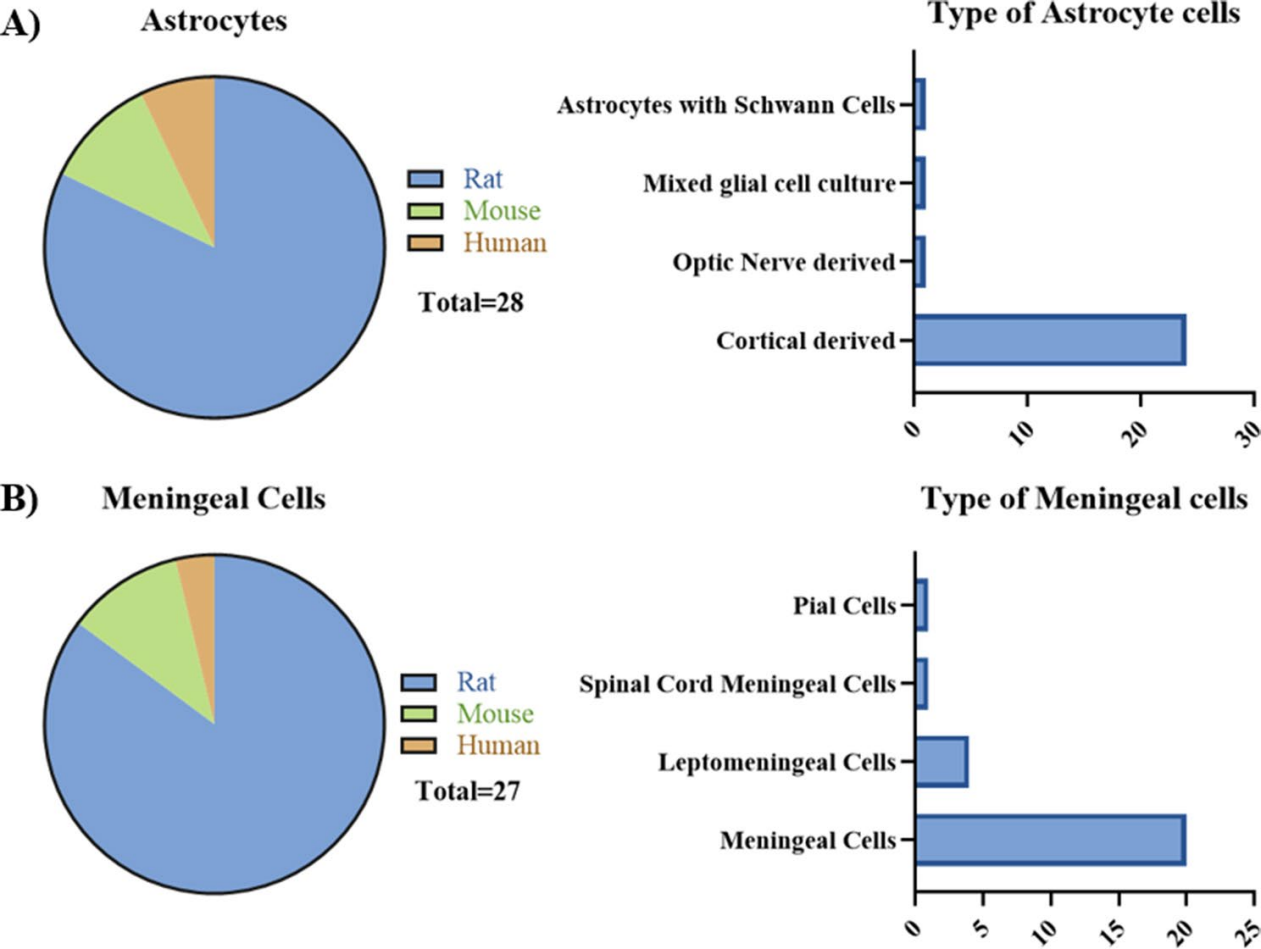
Breakdown of the cell types used in the reviewed papers. **A**) Species and source of the astrocytes. **B**) Species and source of the meningeal cells

The meningeal cells used across these studies were all primary meningeal, leptomeningeal, or pial cells (Fig. 3). They were obtained from newborn rats (20/27), adult rats (3/27), newborn mice (3/27), or adult human (1/27). Nine studies did not specify the strain of rat or mouse (9/27), while others used Wistar rats (5/27), Sprague Dawley rats (8/27), Fischer rats (1/27), or C57BL/6 mice (2/27).

For the astrocytes, there was more variation in the source of the cell (Fig. 3). The cells were obtained from newborn rats (20/27), newborn mice (3/27), adult rats (3/27), human astrocytoma cell line (1/27), or adult human (1/27). Most studies used cortical astrocytes (24/27), whereas two papers (2/27) had other cell types involved. This included a mixed glial cell culture and astrocytes mixed with Schwann cells.

#### Type of co-culture models

There was various co-culture methods used in the included studies from direct cell contact to set ups that only allowed for specific communication (Fig. 2).

Direct co-culture was the most common method utilised (18/27), with direct seeding of one cell on top of the other [14, 16, 38–40, 44–48, 50–57]. Three papers (3/27) also included a simple coverslip method where the meningeal cells and astrocytes were seeded on opposite sides of the coverslips and allowed to grow towards each other [48, 58, 59]. Three papers (3/27) used the Transwell/Millicell model which is a method of in-direct co-culture [55, 57, 60]. Astrocytes were seeded on the bottom of the well while the meningeal cells were seeded on the porous membrane, enabling molecular diffusion without direct cell contact [55, 57, 60]. The majority of the papers did not mention if the meningeal cells were seeded on top of the astrocytes or vice versa [16, 38, 44, 46, 50, 52, 53, 55, 61]. Whereas other papers specified that astrocytes were seeded on top of meningeal cells [14, 45, 48, 51, 54] or that the meningeal cells were seeded on top of astrocytes [39, 40, 56, 57].

**Fig. 4:**
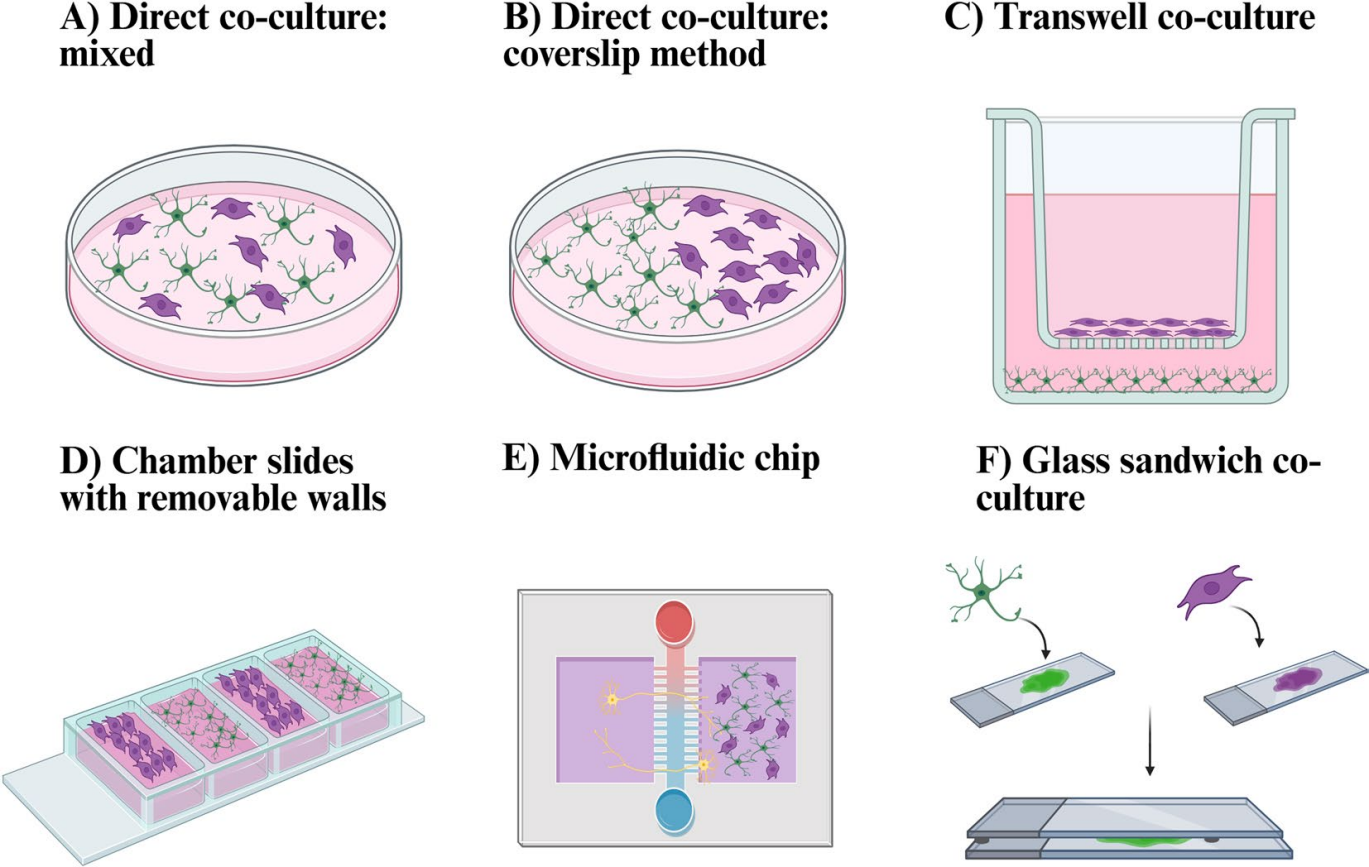
The different co-culture methods which were used in the papers included in this systematic review. **A**) Direct co-culture– mixed. This model directly seeded meningeal cells and astrocytes directly on top of each other [14, 16, 38–40, 44–48, 50–57]. **B**) Direct co-culture – coverslip method. This model seeded the meningeal cells on one side and the astrocytes on the other side, allowing the two cell types to grow towards each other [48, 58, 59]. **C**) Transwell co-culture. This was used with the meningeal cells seeded on the apical side of the membrane and astrocytes on the base of the wells [55, 57, 60]. **D**) The chamber slides with removable wells. This was used to allow the cell types to grow separately. The chamber walls were then removed, allowing the two cell types to grow towards each other [62–64]. **E**) The microfluidic chip. This was used mainly to examine the neurite outgrowth inhibition by seeded neurons in one side, and the co-culture of meningeal cells and astrocytes in the other side [38, 64]. **F**) The glass sandwich method seeded astrocytes on one glass slide and the meningeal cells on a different glass slide. They were place on top of each other, separated by a gas permeable gasket with media filling the space [49]. Created in BioRender. Reardon, E. (2025) https://BioRender.com/0q6zl5b

One study (1/27) explored a set up called “glass sandwich” where meningeal cells and astrocytes were grown on separate glass slides. The two cell types were not in direct contact but separated by a 1 mm thick gas permeable gasket. Media filled the gaps between the cells [49]. Three studies (3/27) utilised the commercially available Nunc/Lab Tek chamber slides to facilitate their co-culture model [62–64]. This allows for the cells to grow towards each other following the removal of the chamber, allowing for an interface between the astrocytes and meningeal cells to form. Two papers (2/27) utilised a fluidic chip model to examine the inhibitory effect of the astrocyte-meningeal co-culture on neurite outgrowth [38, 64] (Fig. 4).

#### Parameters of co-culture models

The methods to which each paper co-cultured the cells varied and the parameters that each method of co-culture utilized were also inconsistent. Typically, primary meningeal cells and astrocytes require the addition of a substrate on the tissue culture plastic or glassware used [65]. Substrates were predominantly poly-L-lysine coated plates (19/27), some on poly-D-lysine coated plates (5/27), fibronectin (2/27), however, five papers did not mention the substrate (5/27).

Most of the cells were grown in Dulbecco’s Modified Eagle Medium (DMEM) or DMEM/F12 culture medium with either foetal bovine serum/foetal calf serum (FBS or FCS) between 10 and 15%. One paper used serum free medium [54], whereas others reduced or omitted serum during the experimental stages of the studies [39, 40, 56]. Conditioned media from either the astrocytes or meningeal cells were also examined in various papers as an additional objective in the co-culture system. However, the inclusion of conditioned media or not is not the focus of the review. Conditioned media was mainly found to enhance the effects already seen in the co-culture model such as morphological changes [14, 45, 53, 60].

Many papers provided limited details on the seeding densities or ratio between the cell types, an important parameter when developing a co-culture model. Where mentioned, the seeding density varied greatly between the papers. Therefore, it is difficult to make a comparison between the seeding density as the objective of each study was different. However, those who used the chamber slides gave more precise seeding densities [62–64], which would allow for the replication of these experiments. Two papers provided information on different ratios and seeding densities of the two cell types. One study found that the astrocytes always overgrew the meningeal cells, suggesting that the meningeal cells drive the astrocyte proliferation [14]. This supports a second study which seeded ten times the number of astrocytes compared to meningeal cells [47].

### Co-culture model assessments

The details regarding the main methods and co-culture parameters for the included papers has been provided. As mentioned, the papers had varying objectives when using this co-culture model and the main outputs are outlines below. Overall outputs from each paper are included in the supplementary file.

#### Neurite outgrowth

Thirteen research papers (13/27) assessed the role of the co-culture as a barrier to neurite outgrowth with a focus on the glial scar. The meningeal cell and astrocyte co-cultures were used to examine the inhibitory effect of neurite outgrowth in either the “healthy” or the glial scar co-culture. Typically, these studies applied an external factor such as mechanical scratch or chemical addition to mimic the glial scar. The studies seeded neuronal cells atop of astrocyte-meningeal co-cultures and examined various growth metrics such as neuron counts, process lengths, and the number of neurons crossing the interface.

There are differences in the models in the studies listed in Table 1. Some papers used just the co-culture alone (5/27), others added TGFβ-1 to induce the glial scar “cluster” (6/27), one (1/27) examined the scratch assay between the interface, and one (1/27) used mechanical stretch to model the glial scar. Overall, most of the papers found that the co-culturing of meningeal cells and astrocytes led to a significant reduction in the growth of neurons, particularly in the meningeal regions. However, this is not found in two papers, where there is only a significant reduction if there TGFβ-1 is added to promote the glial scar model [38, 64]. Overall, these conflicting results indicate the astrocyte-meningeal co-culture by itself is not an adequate model of the glial scar but requires further injury through either mechanical or chemical stimuli.

**Table 1:** A summary of methods, and outputs from the papers which examined neurite outgrowth inhibition. N/A - not applicable

Ref	Co-culture method	Injury	Astrocytes and Meningeal Cell Type	Neuronal Cell Type	Metric	Outcome	Drug/Treatment
[46]	Direct co-culture	N/A	Both obtained from the cortex of 1-day-old rats	Hippocampal neurons	Neurite length (µm)	Increasing the meningeal cell ratio reduces the neurite growth	N/A
[45]	Direct co-culture	N/A	Both obtained from the cortex of neonatal Sprague Dawley rats	Cerebellar granule cells	Percentage of neurons/neurites present and neurite length (µm)	Co-culture significantly reduced the number of neurons and length of neurites compared to astrocyte only control	N/A
[50]	Direct co-culture	N/A	Both obtained from the optic nerve of adult female Sprague Dawley rats	Embryonic retinal neurons	Only visual through immunocytochemistry	Neurons avoided the meningeal cells but grew on the astrocytes	N/A
[47]	Direct co-culture	N/A	Both obtained from the cortex of neonatal rats	Neonatal Dorsal root ganglion neurons	Percent of axons crossing the interface of the astrocyte-meningeal cell co-culture	Almost all neurons crossed from meningeal regions to astrocytes regions, whereas few neurons growing on the astrocytes crossed to meningeal cell regions.	Anti-NG2/anti-NP2, forskolin, IBMX, NT-3, rolipram, and C3-05 all increased the percentage of axons crossing from astrocytes to meningeal cells.
[39]	Direct co-culture	Mechanical stretch	Both obtained from the cortex of newborn rats	Dorsal root ganglion neurons, spinal cord neurons, and cortical neurons	Neurite length (µm) - largest and overall	Co-culture reduced the neurite growth compared to astrocyte control. Astrocyte with mechanical stretch and co-culture with mechanical stretch also reduced neurite outgrowth compared to astrocyte control.	N/A
[63]	Nunc chamber slide	TGFβ-1	Both obtained from the cortex of 1-2-day-old Sprague Dawley rats	Cerebellar neurons	Neurite length/number of neurons	Significantly reduction in the neurite outgrowth in the co-culture in the presence of TGFβ-1.	N/A
[62]	Nunc chamber slide	TGFβ-1	Both obtained from the cortex of 1-2-day-old Sprague Dawley rats	Cerebellar neurons	Neurite length (mm/mm2)	Neurite outgrowth not measured outside of drug treatments.	ChAC/ABC significantly improved the neurite length compared to the co-culture in the presence of TGFβ-1
[56]	Direct co-culture	N/A	Both obtained from the cortex of newborn rats	Postnatal cerebral cortical neurons	Percent of neurites and neurite length (µm)	Co-culture significantly reduced the number and length of neurites compared to astrocyte only control.	Olfactory ensheathing cells improve the neurite growth and length in the co-culture
[59]	Direct- Coverslip method	TGFβ-1	Both obtained from the cortex of P0-2 neonatal Wistar rats	Embryonic cortical neurons	Neurite length (µm)	Neurite outgrowth not measured outside of drug treatments.	DFO improved the neurite outgrowth on co-culture with TGFβ-1.
[44]	Direct co-culture	TGFβ-1	Both obtained from the cortex of P0-P2 C57BL/6 mice	Cortical Neurons	Neurite length/neuron (%)	Neurite outgrowth not measured outside of drug treatments.	Salubrinal improved the neurite outgrowth in the presence of the TGFβ-1.
[38]	Microfluidic chip	TGFβ-1	Both obtained from the cortex of 1-day-old Sprague Dawley rats	Ventral spinal cord 4.1 motor neurons	Length of neurons (µm)	No significant reduction in the neuron growth when in the presence of the co-culture, but significant reduction when TGFβ-1 was added.	siRNA significantly improved the growth of the neurons in the co-culture with TGFβ-1 but no change in co-culture without TGFβ-1
[58]	Direct co-culture	TGFβ-1	Both obtained from the cortex of 2-day-old Wistar rats	Cerebellar granule cells	Neurite length (µm)	Significant reduction in the neurite outgrowth in the co-culture in the presence of TGFβ-1.	ChABC significantly improved the neurite length compared to the co-culture in the presence of TGFβ-1.
[64]	Microfluidic chip	Scratch	Both obtained from the cortex of postnatal day 1 Sprague Dawley rats	Ventral spinal cord 4.1 motor neurons	Length of neurons processes (µm)	No significant reduction in the neuron growth in the presence of the co-culture, but significant reduction in scratch injury.	siRNA significantly improved the growth of the neurons in the co-culture with scratch but no change in normal co-culture

#### Morphology

**Fig. 5:**
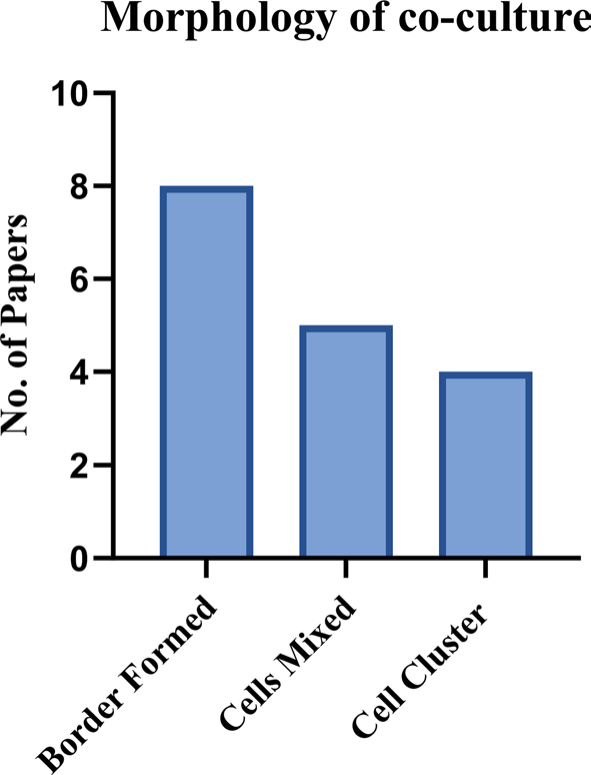
A graphical representation of the morphology of the co-culture across 17 different papers. A border was found (8/17) which is similar to the brain-meninges interface/glia limitans, the cells mixed (5/17) with no distinct borders, and a cell cluster was formed (4/17) in the presence of TGFβ-1

Many studies have examined the astrocyte-meningeal co-culture to address various research questions, often examining the morphological changes (17/27) (Fig. 5).

Many papers found that when the two cells were directly co-cultured, they did not mix but formed a clear boundary resembling the glia limitans (8/17). Overall, when looking at the changes to the morphology of the two cell types, most papers only looked at the changes to the astrocytes. These conclusions were consistent where studies found that the astrocytes, when co-cultured with meningeal cells, were more elongated and slender in shape with finer processes compared to when in monoculture [14, 16, 39, 45].

In contrast, other studies found that there was no interface formed but the two cell types mixed (5/17). Other papers looked at the co-culture model in the presence of TGFβ-1 to examine the glial scar to mimic the in vivo structure and composition such as expression of the correct markers and inhibition of neurite growth. This led to the formation of a cell cluster which is described as similar to the glial scar or fibrotic scar. One study found the addition of TGFβ-1 led to increased proliferation of the meningeal cells but not astrocytes [63]. Another study found a cell cluster formation, however this was in the presence of Schwann cells in the co-culture and not TGFβ-1 [53].

In contrast, other papers had different results to this with either the cells not making any sort of boundary or glial scar cluster. Four papers found that the astrocytes formed circular structures surrounding patches of meningeal cells [39, 40, 56, 66]. Finally, two papers focused more so on the individual cells rather than cluster formation or border formation between the two cells, therefore, limiting the morphological analysis [45, 50]. These conflicting results can be due to a variety of reasons when reviewing each study separately but the inconsistencies in methods, cell type, and seeding densities make it difficult to determine the explicit reason for such conflicting results. This further demonstrates the need to establish a baseline requirement for information to be provided when developing an in vitro co-culture model of the brain-meninges interface or glial scar.

#### List of protein and ECM markers investigated

The majority of the papers (22/27) analysed protein markers or extracellular matrix proteins. Most commonly GFAP was used as an astrocyte marker. However, there is not a consensus on a suitable meningeal cell marker in these identified papers with extracellular matrix proteins being primarily used to identify the cells such as fibronectin, laminin, and collagen. This is a consistent finding throughout various papers with it being difficult to find a specific marker for the meningeal cells. Recent transcriptomic papers have revealed more specific markers for the subtypes of the meningeal cells which could be used in future studies [10, 11, 43, 67, 68]. In Table 2, the main markers used for the meningeal cells, astrocytes, or the ECM have been listed, but only based on the studies that have been included in the PRISMA approach.

**Table 2:** Summary of the main proteins identified and examined in the reviewed papers

Marker	Definition	Ref.	No. of papers	ASC/MC/ECM Marker
**Glial Cell Markers**				
GFAP	Glial fibrillary acidic protein (GFAP) is an intermediate filament protein which is highly expressed in astrocytes [69].	[14, 16, 38–40, 45, 47, 48, 50, 53–57, 59, 60, 62–64]	19	ASC
Aqp4	Aquaporin4 is a water channel present on astrocytic end feet [40]	[40]	1	ASC
A2B5	A2B5 is used as a glial progenitor cell marker for astrocyte progenitor cells, oligodendrocyte precursor cells, and neural stem cells [70].	[14]	1	N/A
S100β	S100β is a calcium-binding protein which is highly expressed in astrocytes [71].	[39]	1	ASC
**ECM Markers**				
Fibronectin	Fibronectin is an extracellular matrix glycoprotein [72] and is present in the basal lamina at the interface between the brain and meninges [61]	[16, 38–40, 47, 48, 50, 53, 55, 56, 59, 60, 62–64]	15	MC/ECM
Collagen IV/I/Collagen1a1	Collagen is a major component of the extracellular matrix and is present in the meninges [73]	[55, 59, 63, 64]	4	ECM
Tenascin-C	Tenascin-C is an extracellular matrix protein [74] and has been shown to be highly expressed in CNS injury such as in glial scar and in optic nerve transection [48].	[39, 45, 48, 50, 55, 59, 63]	7	ECM
Laminin	Laminins is an extracellular matrix glycoprotein [72] and is highly expressed at the brain-meninges interface.	[16, 47, 50, 63]	4	ECM
**Chondroitin Sulfate Proteoglycans (CSPGs)/Glycosaminoglycans (GAGs)**				
Phosphocan	Phosphocan is a CSPG which is a component of the ECM in the brain and meninges.	[39, 44, 47, 57, 59, 63]	6	ECM
Neurocan	Neurocan is a CSPG which is a component of the ECM in the brain and meninges.	[39, 44, 47, 57, 59, 63, 64]	7	ECM
NG2	NG2 is a CSPG which is primarily expressed by oligodendrocytes in the brain	[47, 50, 59, 63, 64]	5	ECM
Dsd-1 proteoglycan	A proteoglycan with chondroitin sulfate chains.	[50]	1	ECM
Versican	Versican is a CSPG which is a component of the ECM in the brain and meninges.	[47]	1	ECM
Brevican	Brevican is a CSPG which is a component of the ECM in the brain and meninges.	[44]	1	ECM
Dermatan sulfate	Dermatan sulfate is a GAG which is part of the ECM.	[62]	1	ECM
Chondroitin sulfate	Chondroitin sulfate is a GAG which is part of the ECM.	[44, 45, 47, 50, 62, 63]	6	ECM
Keratan sulfate	CSPG is a GAG which is part of the ECM.	[50]	1	ECM
**Adhesion Molecules**				
Βeta-1 integrin	Integrins are important in cellular adhesion, migration, and neurite outgrowth [75]	[45]	1	Both
NCAM	Neural cell adhesion molecule (NCAM) is part of the immunoglobulin family and is an adhesion molecule in neurons and glial cells [76].	[50]	1	ASC
N-Cadherin	N-cadherin is part of the calcium-dependent adhesion molecule family and is involved in structural integrity and adhesion [77] and was only found in astrocytes and not in meningeal cells [47].	[47]	1	ASC
Connexin43	Connexin43 is a gap junctional protein which is important in cell-cell communication [78], present in both meningeal cells and astrocytes [79].	[79]	1	Both
**Others**				
SEMA3A/C	Semaphorins are a family of extracellular signalling proteins. Sema3a/3c are inhibitory axon guidance molecules [80] present in the glial scar [47].	[47, 59, 63]	3	MC
Ephb2/EphrinB2	EphB2 is a receptor tyrosine kinase with ephrinB2 is the ligand. This interaction is important in cell signalling, adhesion, and glial scar formation [81].	[38, 59, 63, 64]	4	Glial scar
Raldh2 (Aldh1a2)	Raldh2 is an enzyme involved in retinoic acid synthesis, and was used as a meningeal marker [12]	[47]	1	MC
Did not examine any protein or ECM markers		[46, 49, 52, 58]	4	

ASC – astrocyte marker, MC – meningeal cell marker, ECM – extracellular matrix marker, N/A- not applicable to either specifically

Determining a set of standard markers to analyse is not the focus of this review but rather a discussion on the various markers that were analysed in the studies. Many of the papers examined different proteins, extracellular matrix markers, and pathways which are altered in co-culture compared to monoculture. The following subsection will delve into the various markers used related to protein and ECM analysis. Table 2 outlines many of the proteins and ECM markers which were looked at in the reviewed papers. Many papers also compared and contrasted proteins of interest comparing in co-culture to monoculture, or in the presence of an injury or drug.

#### Protein and ECM comparison

In this section, we review the changes in the aforementioned markers found in the 27 studies. A significant reduction in the expression of laminin in co-culture was found compared to astrocytes alone, but no changes to β1-integrin or tenascin-C. However, there was redistribution of the tenascin-C to the cell borders in co-culture [45]. Other work found that the presence of meningeal cells led to a significant reduction in the collagen expression by astrocytes [55]. There was an increased expression of GFAP found particularly at the border of the meningeal cells [14]. Another study also saw this increase in GFAP, along with a significant increase in phosphocan, neurocan, and tenascin-C expression in co-culture compared to astrocytes alone [39].

There were other experiments conducted within these papers which includes meningeal cell migration, astrocytic assemblies, calcium signalling and more which is mentioned briefly in the supplementary file. Additionally, various papers also compared the in vitro data to in vivo data which is beyond the scope of this review.

## Discussion

The interaction between astrocytes and meningeal cells in vitro has been examined in this systematic review across 27 different papers, focusing on their use to model the brain-meninges interface, glial scar, and overall, the communication between astrocytes and meningeal cells. The systematic review will discuss the papers reviewed by common methods, results, and discussion points that were found, and we will also highlight some of the open questions that need to be addressed in the field. The ultimate aim of this review is to establish what is known, and unknown, to develop an in vitro model of the astrocyte-meningeal co-culture to mimic the brain-meninges interface.

### Objectives of the co-culture models

One main theme throughout this systematic review is that these cells have been co-cultured to model different aspects of the astrocyte-meningeal interaction. Primarily, the co-culture is used to model the glial scar. However, there are contradicting views on if the co-culture alone is a model of the healthy interface which occurs in the brain [14], or if it is modelling the glial scar [39]. Some authors consider the astrocyte-meningeal co-culture alone as modelling the glial scar, and other studies consider it the healthy interface of the brain-meninges interface or the glia limitans. In contrast, many papers believe that an injury must be added to the co-culture to create the glial scar such as TGFβ-1 [38, 44, 59, 63], physical scratch [64], or mechanical stretch [39]. However, the studies reviewed believe there are many structural similarities between the glia limitans and the glial scar which occurs following injury [47, 82, 83]. Furthermore, the literature suggests that various other cell types are key to the glial scar formation such as macrophages, microglia, and more [84]. Therefore, the use of meningeal cells and astrocytes alone may not be sufficient to fully recapitulate the cellular diversity of the glial scar.

Moreover, validation of these models should be conducted before considering it a healthy interface or a glial scar model. This also further emphasizes the necessity of developing a standardized approach for co-culturing astrocytes and meningeal cells.

### Co-culture methods

#### Cell types used

##### Type of co-culture methods

Direct co-culture is the most simplistic co-culture method, which is easy to set-up and inexpensive. Direct co-culture allows for cell contact; however, it cannot differentiate the effects from cell contact versus indirect contact [85]. Furthermore, the model requires a cell medium which is suitable for all cell types which can require optimisation [85]. In the context of the studies reviewed, some researchers seeded the cells on top of each other [14, 16, 38–40, 44–48, 50–56], and others seeded the cells on opposite sides of the well to allow them grow together [48, 58, 59].

Each of the direct co-culture methods reviewed have their own advantages and disadvantages. Regarding the brain-meninges interface or glia limitans, it could be argued that culturing the cells on opposite sides and allowed to grow together is more relevant as this is more so how it would occur in vivo with the pial cells lining the glia limitans [15]. Allowing them to grow towards each other is more beneficial when examining the boundary formed, and the possible development of the shared basement membrane [86]. However, the use of the direct mixed co-culture is more suitable for use in metabolic and viability assays.

In regard to modelling the glial scar, this depends on what type of scar the researchers are interested in. If it is a penetrating wound, the meningeal cells may invade the brain causing the formation of the glial scar due to astrocyte interaction [87]. However, the glial scar can also form in the absence of meningeal cells such as in stroke and is usually modelled using an astrogliosis model [87]. Overall, both direct co-culture models offer valuable insight for different research questions.

Millicell/Transwell inserts are commercially available cell culture inserts which is more expensive than the direct co-culture methods. One benefit of this model is that various configurations of co-culture can be examined: direct, in-direct, multiple cell types. Another advantage to the use of these systems is that transepithelial/endothelial electrical resistance (TEER) can be measured when the cells are separated into two wells or chambers. TEER is a quantitative measure of electrical resistance across a cellular monolayer to examine the permeability and integrity [88], which has been used to measure barrier functions [88]. As the meninges has barrier properties, this is particularly useful, however, none of the groups looked at this in their co-culture models [55, 57, 60] and is an open question to address that some other studies have attempted with monocultures of meningeal cells [65, 89]. Despite the pial-glial boundary/brain-meninges interface being a leaky barrier, it would be useful to measure the TEER as it restricts the passage of immune cells and erythrocytes and would allow for comparison to other brain barriers such as the arachnoid barrier and blood-brain barrier [4, 8, 90, 91].

Two research groups looked at the use of microfluidic chips to examine the neurite outgrowth through the microchannels when there is the presence of the co-culture modelling the glial scar [38, 64]. This is a more complicated set up and more difficult to obtain data or visually see the neurite outgrowth. Chamber slides are used as a more advanced version of separately seeding the cells on opposite sides of the well [62–64]. This allows for more control over the cells and removal of the chamber at the correct timing to allow the cells to meet and form the interface. Finally one group used a glass sandwich co-culture method and is more complicated to set up through seeding cells individually on glass slides [49]. However, one benefit is that the cell types can be analysed separately for further experiments, like the Transwell model, which can be particularly useful if one cell type is of primary interest.

Overall, each of these co-culture models has different advantages and disadvantages dependent on the research question under examination. In order to best model the brain-meninges interface or glia limitans, the use of the chamber slides, coverslip method, and Transwell inserts are the most physiologically relevant when mimicking the in vivo characteristics [35]. However, direct co-culture can allow for many method validation questions to be understood such as the cell seeding density, cell ratio, media composition for the two cells, and to simply examine their interaction together. However, if modelling the glial scar, this varies further dependent on the specific injury. If it is a penetrating which would lead to the meningeal cells invading the brain, the chamber slides or coverslip method could model this however, some type of injury or chemical stressor should be added to suitably model this. If modelling a wound that may penetrate further into the brain, the direct co-culture model with an injury, such as TGFβ-1, could be used. Overall, the models should mimic what we see in vivo and have suitable validation methods to confirm the model replicates the interface.

#### Parameters of co-culture models

Furthermore, the different approaches used in the culturing of the cells can have unwanted effects. Most of the papers used media containing 10–15% serum which has been shown to induce reactivity in astrocytes [92, 93]. This is a major caveat in the studies, particularly studies aiming to model the healthy interface, and overall, can confound the results if the serum effects are not taken into account [94]. Some studies used stepwise serum withdrawal in the astrocyte culture prior to co-culturing reducing the confounding factors [39, 40, 56]. Alternative methods can be used to isolate the cells such as immunopanning which allows for serum free culturing of cells, however, has its own limitations [33]. Overall, we recommend either using lower serum concentrations, including a serum-free control, and/or validating the phenotypes of the cells to elucidate the effects from the serum versus the effects of the co-culture.

In the included studies, most astrocytes were derived from the cortex, with two studies that included other glial cells as well [16, 53]. Similarly with the meningeal cells, the astrocytes have regional heterogeneity depending on their location in the brain, age, sex, and disease state [69, 95–97]. Most of the papers isolated the cells from the cortex, which would be suitable for the glial scar modelling. However, new research shows the glia limitans is made up of specialised astrocyte phenotype with an atypical morphology compared to cortical astrocytes with differential markers such as Myocilin [15, 98]. Further validation of the co-culture model using suitable markers, such as Myocilin for the glia limitans or brain-meninges interface, is recommended. Additionally, in vitro astrocyte cultures often differ in morphology and functional relevance, often not being representative of the complex nature which is present in vivo [99, 100]. Therefore, optimising the culturing protocols to ensure the correct astrocyte phenotype, subsequent validation is necessary for modelling. This has driven the research into iPSC derived astrocytes which may better recapitulate the heterogeneity and complexity seen in vivo [101].

There is limited detail provided on the meningeal cells used in the reviewed papers and often being described as “meningeal cells” or “meningeal fibroblasts” despite there being three distinct layers and a wealth of cellular diversity in the meninges [7]. Only six papers described where the cells were obtained from, either the pial layer [54], the spinal cord [64], or the leptomeninges [45, 48, 51, 79]. It would be beneficial to determine which layer of the meninges the cells were isolated from, however this can be challenging due to difficulty in separating the arachnoid and pia mater [102]. Using the recently defined markers of the pial cells and arachnoid cells [10, 11, 68], this could be used in future studies to better characterize the cell types being used as they have different functions and will therefore interact differently with the astrocytes [11]. Furthermore, regarding the brain-meninges interface, only the pial cells will be in close contact with the astrocytes, and therefore, are the meningeal cell type which should be used for these experiments.

#### Inconsistencies in reporting co-culture method parameters

Based on this systematic review, there is a lack of information provided on the methodological details such as seeding densities, ratios for the cells, or specific co-culturing methods. The lack of information provided on these key parameters is an issue in the research field often leading to a lack of reproducibility [103]. This could explain some of the conflicting and contradicting results between papers. Promisingly, there is a trend in the newer research papers providing significantly more information, particularly in the chamber slides and microfluidic chip studies [38, 64], which will aid in future studies to establish a set of standard parameters for a comparable co-culture method.

Of these methodological parameters, cell viability, cell seeding density, and a timed cell ratio analysis (i.e. ratio of astrocytes to meningeal cells on day 0 vs. day 7) should be examined in all co-culture studies. Throughout these studies, only two papers examined the cell viability, but this was only examined as part of an experimental condition on the co-culture [44, 60]. Furthermore, only two papers analysed the effect of different cell ratios [14, 46], which is a key factor to investigate particularly in the mixed direct cell culture as the seeding ratio of cells does not always stay the same over time [104]. Furthermore, some cells may be more proliferative and therefore, take over the co-culture which should be controlled for. This was shown where the co-culture was overgrown by astrocytes at the late stages of the experiment and was partially due to the seeding density [14]. Examining the astrocyte-meningeal cell ratio could be done using immunostaining or flow cytometry [104, 105].

### Co-culture model assessments

#### Neurite outgrowth

One of the primary outputs from the reviewed papers was effect of the co-culture on neurite outgrowth. The examination of neurite growth is a standard assessment to demonstrate that the co-culture modelled the glia limitans as well as the glial scar with contradicting results. Firstly, some papers found that there was a significant reduction in neurite growth in the co-culture alone [39, 45–47, 50, 56]. Next, some papers found there was no significant reduction in the presence of the co-culture but only when an injury was introduced such as scratch [64], or TGF-β1 [38, 58, 64]. Finally, some papers only examined the improved neurite outgrowth when drugs were added [44, 59, 62]. A consistent finding amongst these papers is that meningeal cells provide an unsuitable substrate for neurite outgrowth compared to astrocytes which is unsurprising based on the literature [106]. This contrasting evidence could be due to various reasons. A more comprehensive validation of the models could allow for a correct annotation of the model which could explain these results. Furthermore, there is a lack of detail on the methods provided which limits comparison between the outputs of the papers [103]. Finally, dependent on the subtype of meningeal cell isolated, this could affect the results due to their different phenotype and function [8]. Therefore, the same suggestion of increasing detail of the methods in the studies is required.

#### Morphology

Many papers examined the cell morphology of the astrocyte-meningeal co-culture with conflicting results across the papers. Firstly, some papers showed that the direct co-culturing of meningeal cells and astrocytes created a border between the cells and did not mix, resembling the glia limitans [14, 16, 47, 48, 55, 59, 63, 64]. Two papers did not find a cellular border formation but instead that the two cell types mixing [39, 40]. Other papers introduced TGFβ-1 to the co-culture and found a “cell cluster” being formed which they reference to being a glial scar [38, 59, 62, 63]. Finally, two papers focused more on individual cell changes [45, 50].

Furthermore, the papers showed that the astrocytes were more elongated in the presence of the meningeal cells, with no papers specifically mentioning the change in morphology of the meningeal cells. This change in morphology of the astrocytes is difficult to explain without functional assessment, as astrocyte morphology cannot be directly correlated with their function or their impact on other cells types [69]. Further evaluation on the changes in gene expression or more functional assessment could unveil interesting findings [107] in more granular, piecemeal method would help the field understand this relationship better. For example, a co-culture model that only allows processes to communicate or for only secretory communication. Overall, this highlights the inconsistent findings between the papers despite similar methods. Again, it would be beneficial to better characterize the models to ensure what they are modelling, be it the glial scar or the brain-meninges interface.

#### Protein and ECM markers

Protein expression analysis is an important aspect for verifying and examining any co-culture. In particular, cell specific protein expression markers are crucial for investigating the health, viability and performance of the co-culture. For astrocytes, GFAP is the most used marker for astrocytes, along with astrocyte reactivity in the glial scar. However, it would be recommended to combine this with another astrocyte marker to sufficiently encapsulate the astrocyte heterogeneity and also be able to better visualise their processes such as aquaporin4 [69, 108]. The glia limitans is mentioned throughout these papers, however, once again it is mentioned regarding both a healthy and injured diseased state. An improved understanding of the heterogeneity of astrocytes would be needed to examine if they are in their healthy or diseased state [97]. Therefore, examining this in vivo and comparing to in vitro co-culture models could allow us to better understand what is occurring at the interface between these cells.

Astrocytes have well established cell-specific markers, but that is not the case currently for meningeal cells. Fibronectin has been used throughout as a marker of meningeal cells, however, it is present in astrocytes [109–111], and other cell types [112]. Therefore, using this as a specific marker of the meningeal fibroblast cells is not the most suitable. This is a consistent issue in the field due to the lack of consensus on the cell types present in the meninges [8]. This is often due to the complex nature of the cells where they have epithelial, fibroblast, and endothelial properties, making them more difficult to characterise [8, 65]. As mentioned, recent meningeal layer specific markers have been identified through transcriptomic analysis including S100a6 for the pia, and Crabp2 for the arachnoid which has been shown to be conserved in both the mouse and human [11]. Different markers were found identifying Icam1 and Slc28a2 as markers of the leptomeninges [113], and Lama1 and Col15a1 for the pia [68]. However, in the studies included in this systematic review, ECM markers which are widely expressed in many cell types, are primarily used as specific markers of the meningeal cells. Therefore, using these recently discovered markers in these studies could further validate the cell type being used [10, 11, 68, 113, 114]. The characterising of these cells in an in vitro co-culture system to verify the expression of these discovered markers is imperative to correctly study the complex interfaces between the brain and the meninges.

Another issue which arises due to the cellular heterogeneity within the meninges is the multiple names being used for these cell types when being examined in vitro such as pial cells, meningeal cells, meningofibroblast cells, leptomeningeal cells, arachnoid cells, fibromeningeal cells, and more [8]. This variety in cell type makes the literature difficult to navigate, which could lead to a loss of studies being identified. Furthermore, these papers do not provide any validation on the cell types being used through protein or gene analysis. These markers have only been recently identified and therefore, many of the papers were published prior to this information being found. Therefore, a recommendation for future studies is to investigate these newly discovered markers in their experiments as a way of identifying and confirming the cell type. However, agreeing to a consensus nomenclature on the meningeal cells and subtypes will be a challenging task [8].

Currently the field of meningeal research is primarily in vivo mouse models which has provided us a wealth of information. The in vitro studies of the meninges, and specifically the interface between the brain and the meninges, is far behind that of the blood-brain barrier and even other cortical-based cell barriers. A combination of in vitro and in vivo studies is needed to drive forward the research as in vitro models can be very useful in asking more specific questions which may not be possible in mouse models [36]. Furthermore, there is a lack of human-derived cells used in the identified studies. This is for a variety of reasons such as the lack of available post-mortem tissue, and the easier access of astrocytes and meningeal cells from mice or rats [115]. However, in other fields, there has been a drive to use humanised cell lines or primary human cells [116]. Having a humanised model of this interface could be key for bridging the gap between mouse and human and better understand the interplay between meningeal cells and astrocytes.

### Other models of the brain and meninges

Outside the scope of this systematic review, there are models which examine the interaction between the meninges and the brain such as spheroids, organoids, and fluidic models. This trend toward advanced in vitro models is only beginning to develop in the research of the brain-meninges interface. As previously mentioned, the meninges are often studied at the developmental stage rather than the adult stage as they have been shown to have important roles in the development of the brain [11]. One study used a cortical organoid model using three induced pluripotent stem cell (iPSCs) cell lines [117]. This organoid model was then encapsulated using commercially bought human meningeal cells to examine the effects on corticogenesis and brain development. Overall, the meningeal cells allowed for improved laminar organisation of the organoid, along with enhanced radial glia and astrocyte formation, therefore, highlighting the importance of the meninges in in vitro models of the brain and CNS development [117]. Furthermore, a recent study generated a leptomeningeal neural organoid fusion model, which is comprised of a neural organoid derived from human iPSCs which are fused with embryonic mouse leptomeninges [115]. This model allows for the investigation of the brain-meninges signalling, highlighting the increasing interest in the bi-directional signalling at this interface.

Cerebrospinal fluid (CSF) flow in the meninges should also be introduced to in vitro models, which could better recapitulate the in vivo environment [118]. This was done in one study which described a 3D perfusion bioreactor-based 3D model of the subarachnoid space using primary human meningeal cells. Under physiological conditions, there is consistent CSF flow which is tightly regulated and consistent. This has been shown to be important in the maintenance of the meningeal state and functions of the meningeal cells as shown through RNA-sequencing and gene ontology analysis [119]. Overall, there is a growing interest in the importance of the bi-directional signalling between the brain and the meninges through use of in vitro models.

### Clinical and therapeutic importance

This systematic review highlights the lack of in vitro models of the brain-meninges interface using astrocyte-meningeal co-cultures. However, it also highlights the various ways through which astrocytes and meningeal cells interact such as inhibiting neurite outgrowth, alterations in morphology, along with changes in gene and protein expression. Improved modelling of this barrier could have important implications in CNS therapeutics due to the recent interest as an alternative route for drug delivery [27, 35]. Such an in vitro model would allow for drug screening of potential therapeutics in more personalised and humanised models [35, 120]. Additionally, this model could be important in studying the barrier changes in both neurodevelopment, neurodegeneration, and disease as it is evident this bi-directional signalling is important in healthy and diseased states [23].

### Future perspectives

Based on the current literature, there are some key areas which require further exploration. Currently, there is no published paper to derive leptomeningeal cells or glia limitans specific astrocytes from iPSCs. This has hindered the development of advanced brain-meningeal iPSC models where neural organoids were developed, however, has to use primary human or mouse meningeal cells [115, 117]. Future work could look at deriving these cells from iPSCs which could include inducing the vast fibroblast and astrocyte subtypes, along with inclusion of other important cells which would improve the relevancy of these models [121]. Furthermore, many of the included papers use simple 2D culture with little complexity. Recent work highlights the importance of correct substrate stiffness [122], ECM [119], CSF flow dynamics [119], and a 3D environment [99, 119] in meningeal or astrocyte models. We believe that inclusion of these bioengineering aspects along with the use of iPSC derived cells is key to a more mimetic model of the complex brain-meninges interface. Furthermore, we recommend further characterisation of this interface in vivo and validation of the models.

## Conclusion

This systematic review is the first to underscore the potential of the astrocyte-meningeal co-cultures to model the different CNS interfaces such as the brain-meninges interface, glial scar, optic nerve, and spinal cord. However, it also reveals critical shortcomings within the literature. This includes a lack of consistent methodologies, often with omitted information limiting the reproducibility of these models and leading to contradicting findings. This review recommends the standardisation of methods, detailed protocols to be provided, validation of the model, and use of more advance techniques in future studies which will be essential to improving the reproducibility and relevance of these in vitro models.

## Supplementary Information

Below is the link to the electronic supplementary material.

Supplementary Material 1

## Data Availability

No datasets were generated or analysed during the current study.

## Abbreviations

AQP4: Aquaporin4
BPY: 2,2’-bipyridine-5,5’-decarboxylic acid
cAMP: Cyclic adenosine monophosphate
ChAC/ABC: Chondroitinase AC/ABC
CNS: Central Nervous System
CRABP2: Cellular retinoic acid-binding protein 2
CSPG: Chondroitin Sulfate Proteoglycans
CXCL12: C-X-C motif chemokine 12
DFO: Deferoxamine mesylate
DMEM: Dulbecco’s Modified Eagle Medium
ECM: Extracellular matrix
FBS: Foetal Bovine Serum
FCS: Foetal Calf Serum
GAGs: Glycosaminoglycans
GFAP: Glial fibrillary acidic protein
IBMX: 3-isobutyl-1-methylxanthine
ICAM1: Intercellular Adhesion Molecule 1
iPSCs: Induced pluripotent stem cells
L-PGDS: Lipocalin-type prostaglandin D synthase
NCAM: Neural cell adhesion molecule
NG2: Neural/glial antigen 2
NT-3: Neurotrophin-3
OGD: Oxygen-glucose deprivation
PDL: Poly-D-Lysine
PLL: Poly-L-Lysine
PRISMA: Preferred Reporting Items for Systematic Reviews and Meta-Analyses
RALDH2: Retinaldehyde dehydrogenase 2
S100A6: S100 calcium-binding protein A6
S100β: S100 calcium-binding proteinβ
SEMA3A/C: Semaphorin 3 A
siRNA: Small interfering RNA
SLC28A2: Solute Carrier Family 28 Member 2
TBI: Traumatic Brain Injury
TEER: Transepithelial/Transendothelial Electrical Resistance
TGF β-1: Transforming growth factor β 1
